# Characteristics of the gut microbiota in patients with advanced non-small cell lung cancer who responded to immune checkpoint inhibitors

**DOI:** 10.1038/s41598-025-08049-4

**Published:** 2025-07-02

**Authors:** Hideyuki Komatsu, Takuya Sugimoto, Yuzuru Ogata, Takahito Miura, Masanori Aida, Hiroyuki Nishiyama, Mitsuhisa Kawai, Yukihiro Yano, Masahide Mori, Yoshiyuki Shishido

**Affiliations:** 1 Yakult Central Institute, Yakult Honsha Co., Ltd., 5-11 Izumi, Kunitachi-shi, Tokyo, 186-8650 Japan; 2 Department of Thoracic Oncology, National Hospital Organization, NHO Osaka Toneyama Medical Center, 5-1-1 Toneyama, Toyonaka-shi, Osaka, 560-8552 Japan

**Keywords:** Non-small cell lung cancer, Immune checkpoint inhibitors, Gut microbiome, *Bifidobacterium*, *Levilactobacillus*, Microbial diversity, Non-small-cell lung cancer, Microbiota

## Abstract

Despite the introduction of immune checkpoint inhibitors (ICIs) in the treatment of lung cancer, the number of deaths from lung cancer remains high, and further improvements in response rates are necessary. Recently, the gut microbiota has been reported to be involved in the therapeutic effects of ICIs; however, only a few studies have examined patients with lung cancer in this context. In the current study, we aimed to explore the association between the gut microbiota before therapy and the efficacy of ICIs in patients with advanced non-small cell lung cancer (NSCLC). The a-diversity of the intestinal microbiota in patients who responded to ICI treatment (responders) was significantly higher than that in those who did not respond to ICIs (non-responders). Additionally, the abundance of *Bifidobacteriaceae* was significantly higher in the responders than in the non-responders. Furthermore, patients with a high abundance of *Bifidobacteriaceae* had significantly longer overall survival than those with a low abundance. Counts of *Levilactobacillus brevis* were significantly higher in responders than in non-responders. Our findings suggest that a higher diversity of the gut microbiota and an abundance of *Bifidobacterium* and/or *L. brevis* are distinctive features of the microbiota in patients with NSCLC who respond to ICI treatment.

## Introduction

Approximately 2.2 million individuals are newly diagnosed with lung cancer annually worldwide^1^, and approximately 75% of these patients die within five years after diagnosis^2^. In Japan, approximately 120,000 individuals are diagnosed with lung cancer every year, which is also the leading cause of cancer-related deaths^3^. Despite efforts to improve early detection, many cases have been shown to have progressed to an advanced stage by the time of initial presentation and are not eligible for surgery. Therefore, further advances in detection are necessary for appropriate diagnosis and treatment. The emergence of immune checkpoint inhibitors (ICIs), such as those targeting programmed cell death 1 (PD-1) or its ligand (PD-L1), has transformed the treatment and management of advanced non-small cell lung cancer (NSCLC), which constitutes most lung cancers^1^. Several studies have established the anticancer effects of anti-PD-1 antibodies^4–6^, and PD-L1 expression in tumor tissues has been validated as a companion diagnostic test in clinical practice. Nevertheless, the efficacy of ICI treatment in NSCLC remains insufficient in patients with specific metastases or those requiring retreatment. These facts highlight the need for continued research to enhance the effectiveness of ICI treatment in NSCLC and identify patient subgroups most likely to benefit^7,8^. Identifying biomarkers that can more accurately predict clinical responses and/or developing more effective means of sensitizing tumors to ICI therapy is a pressing need to improve the efficacy of ICI therapy in advanced NSCLC.

In recent years, it has become evident that the gut microbiota plays a crucial role in the response to cancer therapy, including response to ICI treatment, and can be targeted to enhance the efficacy of cancer immunotherapy^9–11^. Studies have confirmed the relationship between specific microbial species and their effects on cancer immunotherapy for various bacterial species^12^. In a clinical study investigating the therapeutic effect of anti-CTLA-4 antibodies on advanced malignant melanoma, patients with *Faecalibacterium* and other Bacillota (formerly Firmicutes) demonstrated longer progression-free survival (PFS) and overall survival (OS) than those with a predominance of Bacteroidota (formerly Bacteroidetes)^13^.

Several studies have revealed a significant disparity in the relative abundance of *Akkermansia muciniphila*, *Alistipes putredinis*, *Bifidobacterium longum*, *Prevotella copri*, *Ruminococcaceae* USG 13, and *Agathobacer* in the gut microbiota of patients with lung cancer who responded to treatment (responders) and those who did not (non-responders). These species are associated with a favorable clinical outcome in patients with NSCLC^14–16^. Meanwhile, in terms of poor prognostic predictors, an increased abundance of *Firmicutes* and *Actinobacteria* phyla, as well as *Bifidobacterium breve* has been reported to correlate with short PFS^17^. Despite numerous studies conducted in this domain in recent years, the results are inconsistent. Several questions remain unanswered regarding the relationship between the gut microbiota and treatment response in patients with lung cancer. The observed inconsistencies in the results may be attributed to individual variations in intestinal flora, which are influenced by the host’s genetic makeup, history of antibiotic usage, and dietary habits. Additional factors contributing to these inconsistencies include geographical differences, racial diversity, variations in sample collection timeframes, reference databases, and study endpoints. Furthermore, technical discrepancies, such as those arising from 16S rRNA sequencing and metagenomic approaches, may also play a role. The functional diversity of the gut microbiota is considered a significant factor as well^17,18^. In particular, we believe that the conventional wisdom that the human gut microbiome differs according to the race and residential area of the study subjects has a significant impact on the inconsistent results^19^. For example, *Bifidobacterium* spp. is common among Japanese individuals^20,21^. It is important to identify unique and original species and the composition of microbiota depending on race and residential area. Focusing on regional characteristics makes it possible to determine a relationship between regional bacterial flora and therapeutic effects. Consequently, we posit that the human intestinal microbiota exhibits significant regional variation, primarily influenced by dietary habits. Common bacterial species beneficial to ICI treatment response are unlikely to be universally identified across different regions and ethnicities. Therefore, instead of pursuing a universal approach to predict intestinal microbiota profiles and ICI treatment response, it may be more pragmatic to identify biomarkers predictive of ICI treatment response by focusing on regions with similar dietary practices and intestinal microbiota profiles.

Therefore, in the current study, we aimed to investigate the association between the gut microbiota before therapy and the efficacy of ICI therapy in patients with advanced NSCLC in Osaka. Stool samples were collected from patients prior to initiation of ICI treatment, and the fecal bacterial content was analyzed. The gut microbiota of responders and non-responders was prospectively compared, and the relationships between specific bacterial groups and response rates to ICIs, antibiotic history, and survival time were examined.

## Methods

### Study design

This single-center study was designed as a prospective observational investigation to explore the relationship between the gut microbiome before therapeutic intervention and the efficacy of ICI therapy in patients with NSCLC.

### Participants

We enrolled patients with a definitive diagnosis of NSCLC who underwent ICI therapy between June 2017 and June 2018 at the National Hospital Organization NHO Osaka Toneyama Medical Center. A total of 23 patients were included in the study, all of whom met the selection criteria after receiving an explanation and providing informed consent. Finally, analysis was performed on 19 cases that submitted analyzable samples. The samples were numbered with experimental ID and then sent to the Yakult Central Institute where microbiota analysis was carried out. Therapeutic effects were diagnosed using the Response Evaluation Criteria in Solid Tumors (RECIST) guidelines (version 1.1), and we defined responders (R) (partial response and stable disease after ICI treatment) and non-responders (NR) (progressive disease after ICI treatment). Information regarding the results of diagnosis and stratification (R or NR) was not disclosed until the data were fixed in the microbiota analysis. After fixing the data in microbiota analysis, the samples and therapeutic effects were connected according to the experimental ID.

### Preparation of stool samples and DNA extraction

Fecal samples from 19 patients were obtained before ICI therapy commenced as per the study’s explanatory documentation. Stool samples were prepared as described by Kubota et al.^22^. Each sample was weighed and diluted tenfold using RNAlater (feces:RNAlater = 1:9). The mixture was then agitated for 10 min at 1048 rpm using a ShakeMaster Auto (Bio Medical Science Co., Ltd., Tokyo, Japan). Subsequently, 200 µL of the suspension prepared from the fecal diluent was added to a 2 mL screw cap tube containing 1 mL of phosphate-buffered saline. The contents were stirred using a vortex mixer and centrifuged at 13,000 × *g* for 5 min at 4 °C, after which 1 mL of the supernatant was discarded. This process was repeated twice. The resulting suspension (200 µL) was stored at –30 °C until DNA extraction, which was carried out as previously described by Matsuki et al.^23^.

### 16S rRNA gene sequence for microbiota analysis

The V1-2 regions of the 16S rRNA gene in each sample were amplified with an ABI PRISM® 7500 Real-Time PCR System (Applied Biosystems, Foster City, CA, USA), using 27Fmod2 and 338R primers, as previously described^24^. Amplified DNA was purified using the AMPure XP kit (Beckman Coulter, Brea, CA, USA). After quantitation using the Quant-iT Pico-Green double-stranded DNA assay kit (Life Technologies Japan, Ltd.), the gene was sequenced on MiSeq system (Illumina, Inc., San Diego, CA, USA) using MiSeq Reagent Kits v2 (Illumina, Inc.), as reported in our previous study^25^. The sequences obtained from the MiSeq platform were analyzed using the open-source software package Quantitative Insights Into Microbial Ecology 2 (QIIME2) (2020.2)^26^, and the SILVA 138 database (https://www.arb-silva.de/) was used to annotate taxonomic information. To account for differences in sampling effort between samples, the Shannon index, Faith’s phylogenetic diversity (PD), and the number of observed features was estimated for 13,000 randomly selected sequences to calculate a-diversities. The sequence count of 13,000 was determined based on the sample with the smallest number of reads, following confirmation of an adequate number of reads by consulting the rarefaction curve.

### Determination of bacterial count by rRNA-targeted reverse transcription quantitative PCR

We used the Yakult Intestinal Flora-SCAN (YIF-SCAN®), a bacterial rRNA-targeted reverse transcription quantitative PCR (RT-qPCR) system, to analyze the composition of the gut microbiota^24–26^. The threshold values of the standard curve in the linear range were used to obtain the corresponding bacterial cell count in each nucleic acid sample, which was used to determine the number of bacteria per sample. The specificity of the RT-qPCR assay was determined using group, genus, and species-specific primers as previously described^27–30^. The primer sequences are listed in Supplementary Table S1^23,27–29,31–36^. The total number of bacterial groups was considered as the total number of bacteria. In the present study, the bacterial counts of 17 of 19 patients were compared. Specimens from two participants were excluded because their feces were poorly preserved and were not suitable for measuring bacterial counts.

### Statistical analysis

Statistical analyses were conducted using R software, v.3.5.2. Fisher’s exact test was used to compare the patient detection rates between the groups. For data with a skewed distribution, the median [interquartile range] was used and the Mann–Whitney *U* test was used for analysis. The number of gut bacteria was logarithmically transformed for analysis. When the microbiota counts were lower than the limit of detection, a value of half the detection limit was assigned for statistical calculation. Statistical significance was established at *p* < 0.05. Cox proportional hazards regression analysis was used to compare disease progression and death between two groups stratified based on body mass index (BMI) and diversity and abundance of *Bifidobacteriaceae*, independently. The patients were divided into the “Yes” group and the “No” group, or the positive group and the negative group based on antibiotic treatment and the detection of *L. brevis*, respectively. Patients with median values of BMI, diversity, and abundance of *Bifidobacteriaceae* were assigned to the “low BMI”, “low diversity,” and “low abundance of *Bifidobacteriaceae*” groups, respectively, because their values were below average. Progression-free survival (PFS) and overall survival (OS) were calculated using the Kaplan–Meier method, and the survival distributions between the two groups, high and low abundance of *Bifidobacteriaceae*, and between two groups, *L. brevis*-positive, and *L. brevis*-negative, were compared using the log-rank test.

### Ethics statement

The Institutional Ethics Committee of Osaka Toneyama Medical Center approved this study (1706-8), and written informed consent was obtained from all patients. The study was conducted in accordance with the Ethical Guidelines for Medical and Health Research Involving Human Subjects and current legal regulations in Japan and the Declaration of Helsinki and its subsequent revisions.

## Results

### Baseline characteristics of the participants

Table 1 shows the pretreatment characteristics of the 19 patients before initiation of ICI therapy. A notable difference was observed in BMI between the R and NR groups, with the former exhibiting higher values. However, no significant differences were observed for any of the other parameters.

**Table 1:** Patient characteristics at baseline.

Variables	R (n = 12)	NR (n = 7)	*p*-value
Sex, male/female	8/4	3/4	0.377
Age (years)^§^	74.0 [66.0, 76.5]	72.0 [68.5, 76.0]	0.832
Smoking status, ex/never	10/2	5/2	0.603
Histology, non-Sq/Sq	7/5	6/1	0.333
BMI (kg/m^2^)^§^	23.2 [22.0, 24.3]	19.3 [18.0, 21.1]	**0.028**
Staging, ≤ III/IV	4/8	3/4	1.000
ECOG performance status, ≤ 1/ ≥ 2	10/2	6/1	1.000
PD-L1 expression, < 50%/ ≥ 50%	4/8	3/4	1.000
0%/ ≥ 1%	2/10	1/6	1.000
ICIs, nivolumab/pembrolizumab	3/9	2/5	1.000
ICI treatment line, first/second or later	8/4	3/4	0.377
PNI^§^	42.8 [38.0, 47.4]	39.5 [37.8, 45.3]	0.472
NLR^§^	3.44 [2.55, 4.15]	4.32 [3.56, 4.76]	0.196
Antibiotics prior to treatment, no/yes^$^	9/3	2/5	0.074
Steroids prior to treatment, no/yes	10/2	6/1	1.000

*R* responder, *NR* non-responder, *Sq* squamous, *BMI* body mass index, *ECOG* Eastern cooperative oncology group, *PNI* prognostic nutritional index, *NLR* neutrophile/lymphocyte ratio.

*p*-values were estimated using the Mann–Whitney *U* test for continuous variables or Fisher’s exact test for categorical variables. ^§^Median [interquartile range].

^$^Patients who received antibiotic treatment for more than four consecutive days within the last 1 month prior to ICI treatment. Patients who underwent bronchoscopy within one month prior to initiating ICI treatment were administered short-term antibiotics to prevent infection, with the maximum duration being four consecutive days. Consequently, cases were categorized into a high exposure intensity group and a relatively low exposure intensity group based on the criterion of receiving antibiotic treatment for four consecutive days. *p*-value entries in bold indicate *p* < 0.05.

### Fecal microbiota measurement

The first step involved conducting a diversity analysis of the two groups of patients. The results indicated that the Shannon index and Faith’s PD were higher in the R group than in the NR group (Table 2). The relative abundance of intestinal bacteria was then compared in the R and NR groups (Table 3, Supplementary Table S2). At the family level, the percentage of *Bifidobacteriaceae* was significantly higher in the R group than in the NR group (Table 3). The PD-L1-positive rate was 16/19 in the participants of the present study (Table 1), and there was no significant difference in the PD-L1-positive rate/ PD-L1-expression rate between the high and low groups of a-diversities and relative abundance of *Bifidobacteriaceae* (Supplementary Table S3). Finally, a comparison was made between the bacterial counts in each group, as determined by RT-qPCR, between the R and NR groups (Table 4). Interestingly, *L. brevis* was detected only in patients in the R group (6/10 patients, p = 0.035) (Supplementary Table S4), and the *L. brevis* counts in the R group were significantly higher than those in the NR group (Table 4).

**Table 2:** Differences in a-diversities in the gut microbiome of R and NR groups.

	R	NR	*p*-value
Shannon index	6.47 [6.11, 6.72]	5.67 [5.50, 5.93]	**0.045**
Faith’s PD	37.1 [33.5, 47.3]	30.3 [26.9, 30.7]	**0.022**
Observed features	405 [330, 519]	314 [247, 364]	0.100

*R* responder, *NR* non-responder, *PD* phylogenetic diversity.

Data are expressed as median [interquartile range].

*p*-values were estimated using the Mann–Whitney *U* test. *p*-value entries in bold indicate *p* < 0.05.

**Table 3:** Differences in relative abundances at the family level by 16S rRNA sequencing in the gut microbiome of R and NR groups.

Family	Relative abundance (%)				*p*-value
	R		NR		
*Bifidobacteriaceae*	2.96	[0.77, 9.83]	0.18	[0.09, 1.22]	**0.045**
*Coriobacteriaceae*	0.62	[0.26, 1.40]	0.42	[0.13, 2.26]	0.800
*Eggerthellaceae*	0.25	[0.15, 0.45]	0.08	[0.06, 0.43]	0.340
*Bacteroidaceae*	19.46	[11.04, 28.71]	30.17	[18.89, 37.98]	0.261
*Barnesiellaceae*	0.37	[0.03, 1.13]	0.00	[0.00, 1.00]	0.306
*Marinifilaceae*	0.33	[0.06, 0.69]	0.37	[0.16, 0.56]	1.000
*Prevotellaceae*	0.68	[0.01, 2.92]	6.28	[0.01, 12.60]	0.650
*Rikenellaceae*	1.31	[0.55, 3.63]	0.79	[0.45, 2.44]	0.592
*Tannerellaceae*	3.83	[1.43, 4.25]	3.06	[2.19, 3.71]	0.902
*Desulfovibrionaceae*	0.11	[0.03, 0.20]	0.11	[0.04, 0.14]	0.553
*Erysipelatoclostridiaceae*	0.35	[0.17, 1.09]	0.35	[0.23, 1.61]	0.536
*Erysipelotrichaceae*	0.57	[0.25, 1.20]	0.72	[0.19, 2.01]	0.773
*Lactobacillaceae*	0.16	[0.01, 0.52]	0.10	[0.01, 0.20]	0.650
*Streptococcaceae*	3.06	[0.78, 9.07]	1.37	[0.60, 4.39]	0.592
*Christensenellaceae*	0.14	[0.05, 0.93]	0.04	[0.01, 0.06]	0.090
*Clostridiaceae*	0.32	[0.02, 1.21]	0.03	[0.01, 0.46]	0.271
*Lachnospiraceae*	18.50	[13.04, 23.54]	24.27	[20.25, 28.43]	0.142
*Monoglobaceae*	0.10	[0.01, 0.12]	0.03	[0.00, 0.19]	0.865
*Butyricicoccaceae*	0.10	[0.05, 0.22]	0.14	[0.06, 0.24]	0.592
*Oscillospiraceae*	2.97	[0.87, 4.05]	0.86	[0.65, 1.11]	0.120
*Ruminococcaceae*	8.37	[4.61, 10.71]	1.94	[1.26, 4.87]	0.056
*Anaerovoracaceae*	0.21	[0.08, 0.36]	0.14	[0.05, 0.30]	0.612
*Peptostreptococcaceae*	0.98	[0.19, 1.64]	0.50	[0.09, 1.67]	0.902
*Acidaminococcaceae*	0.19	[0.01, 0.40]	1.03	[0.00, 1.22]	0.520
*Veillonellaceae*	0.15	[0.02, 0.49]	0.06	[0.01, 2.63]	0.773
*Sutterellaceae*	0.26	[0.00, 0.93]	0.03	[0.00, 0.64]	0.670
*Enterobacteriaceae*	0.37	[0.07, 0.76]	0.62	[0.21, 2.49]	0.711

*R* responder, *NR* non-responder.

Data are expressed as median [interquartile range]. Only families with median abundance above 0.1% are shown.

*p*-values were estimated using the Mann–Whitney *U* test. *p*-value entries in bold indicate *p* < 0.05.

**Table 4:** Fecal counts of the gut microbiota determined by RT-qPCR.

	Fecal bacterial counts (log_10_ cells/g of feces)				*p*-value
	R		NR		
Total bacteria	10.1	[9.70, 10.4]	9.97	[9.82, 10.2]	0.89
*Clostridium coccoides* group	8.84	[8.49, 9.34]	9.36	[9.19, 9.48]	0.32
*C. leptum* subgroup	9.13	[9.02, 9.33]	9.39	[9.08, 9.70]	0.48
*Bacteroides fragilis* group	7.70	[6.75, 8.59]	8.62	[7.99, 8.94]	0.32
*Bifidobacterium*	9.19	[8.48, 9.32]	8.22	[6.98, 9.18]	0.32
*Atopobium* cluster	9.37	[9.00, 9.54]	9.41	[8.41, 9.49]	0.89
*Prevotella*	5.16	[2.50, 6.93]	5.34	[2.50, 7.92]	0.61
*C. perfringens*	2.48	[1.15, 4.21]	1.15	[1.15, 4.19]	0.62
*C. difficile*	Not detected		Not detected		–
Total lactobacilli	7.17	[6.51, 8.14]	6.76	[6.09, 7.42]	0.32
*Lactobacillus*	5.70	[5.01, 7.05]	5.94	[4.60, 6.47]	0.85
(Formerly *Lactobacillus gasseri* subgroup)					
*Levilactobacillus brevis*	2.82	[1.15, 3.74]	Not detected		**0.017**
(Formerly *Lactobacillus brevis*)					
*Lacticaseibacillus*	5.75	[2.16, 5.87]	1.50	[1.50, 4.45]	0.19
(Formerly *Lactobacillus casei* subgroup)					
*Limosilactobacillus fermentum*	6.77	[3.06, 7.89]	5.60	[3.78, 6.99]	0.37
(Formerly *Lactobacillus fermentum*)					
*Fructilactobacillus fructivorans*	1.15	[1.15, 1.15]	Not detected		0.40
(Formerly *Lactobacillus fructiborans*)					
*Lactiplantibacillus*	4.70	[3.29, 5.42]	3.29	[1.96, 4.51]	0.20
(Formerly *Lactobacillus plantarum* subgroup)					
*Limosilactobacillus* (except *L. fermentum*)	5.35	[4.82, 6.46]	4.98	[4.12, 5.61]	0.26
(Formerly *Lactobacillus reuteri* subgroup)					
*Ligilactobacillus* and *Liquorilactobacillus*	1.15	[1.15, 1.15]	1.15	[1.15, 1.84]	0.42
(Formerly *Lactobacillus ruminis* subgroup)					
*Latilactobacillus*	3.55	[1.65, 4.09]	4.04	[1.15, 4.71]	0.88
(Formerly *Lactobacillus sakei* subgroup)					
*Enterobacteriaceae*	6.39	[5.21, 7.55]	5.93	[5.63, 7.47]	0.85
*Enterococcus*	5.52	[2.42, 6.23]	4.58	[3.01, 6.89]	0.92
*Streptococcus*	9.03	[8.50, 9.93]	8.75	[7.47, 9.32]	0.23
*Staphylococcus*	4.33	[3.24, 5.02]	4.13	[2.32, 4.68]	0.56
*Pseudomonas*	Not detected		1.50	[1.50, 2.98]	0.082

*R* responder, *NR* non-responder.

Data are expressed as median [interquartile range]. *p*-values were estimated using the Mann–Whitney *U* test. *p*-value entries in bold indicate *p* < 0.05.

### Influence of antibiotics on the gut microbiota

We examined the impact of antibiotics on the gut microbiota. Specifically, we compared the relative abundance of bacteria between antibiotics prior to treatment (“Yes” group) and non-treatment (“No” group) at the family level. Our findings revealed that the percentage of *Bifidobacteriaceae* was significantly lower in patients in the antibiotic “Yes” group than in the antibiotic “No” group (Supplementary Table S5). Additionally, the count of *L. brevis* was significantly lower in in patients the antibiotic “Yes” group than in the antibiotic “No” group (Supplementary Table S6).

### Analysis of relationships between clinical and microenvironmental parameters of the gut and prognosis

**Fig. 1:**
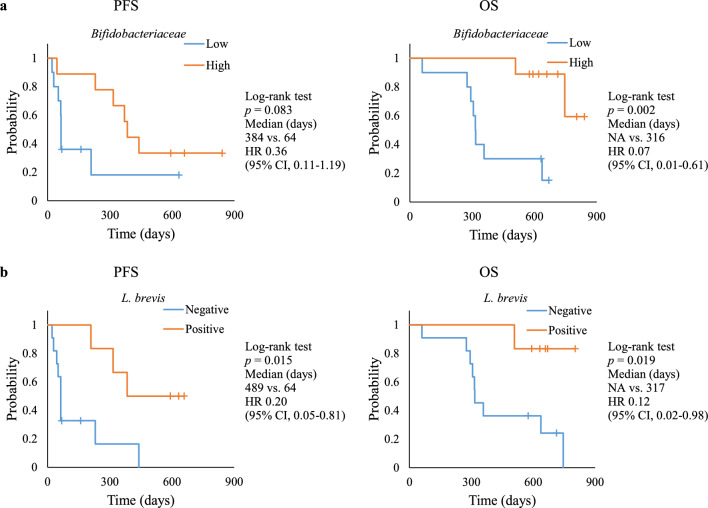
Association between the gut microbiome and response to ICI therapy. Comparison of Kaplan–Meier plot survival curves by a log-rank test. *PFS* progression-free survival, *OS* overall survival, *NA* not available, *HR* hazard ratio, *CI* confidential interval. (**a**) Patients with high and low abundance of *Bifidobacteriaceae*. (**b**) Patients in *L. brevis*-positive and *L. brevis*-negative groups.

Table 5 illustrates the results of univariate analysis of the characteristics of the patients and gut microbiota at baseline for disease progression and death. In terms of disease progression, there was a significant difference in BMI (*p* = 0.044) and counts of *L. brevis* (*p* = 0.024). In the low-BMI group, the risk was higher, and in the *L. brevis*-positive group, the risk was lower. In terms of death, the risk was significantly increased in the antibiotic “Yes” group (*p* = 0.046). However, high diversity indicated by the Shannon index, high abundance of *Bifidobacteriaceae*, and the *L. brevis*-positive group showed lower risk (*p* = 0.022, *p* = 0.015, and *p* = 0.048, respectively). Considering these findings, further analysis was conducted to examine the correlation between the two characteristics of the gut microbiota and prognosis using the log-rank test. The results revealed that the “high group” had longer PFS and OS than the “low group “ in terms of both the abundance of *Bifidobacteriaceae* (Fig. 1a) and counts of *L. brevis* (Fig. 1b).

**Table 5:** Univariate analysis of characteristics of patients and the gut microbiota at baseline for disease progression and death.

Prognostic factors	For progression		*p*-value	For death		*p*-value
	HR	95% CI		HR	95% CI	
BMI, < 22/ ≥ 22	3.19	1.03–9.85	**0.044**	1.48	0.39–5.52	0.563
Antibiotics prior to treatment, yes/no	1.79	0.55–5.81	0.331	4.25	1.03–17.6	**0.046**
Shannon index, high/low	0.59	0.20–1.78	0.351	0.16	0.03–0.77	**0.022**
Faith’s PD, high/low	0.75	0.25–2.24	0.602	0.51	0.14–1.85	0.308
*Bifidobacteriaceae*, high/low	0.36	0.11–1.19	0.094	0.07	0.01–0.61	**0.015**
*L. brevis*, positive/negative	0.20	0.05–0.81	**0.024**	0.12	0.02–0.98	**0.048**

*HR* hazard ratio, *CI* confidence interval, *BMI* body mass index, *PD* phylogenetic diversity.

*p*-values were estimated using Cox proportional hazards regression analysis. *p*-value entries in bold indicate *p* < 0.05.

## Discussion

The objective of our study was to establish a connection between the gut microbiota prior to therapy and the efficacy of ICIs in patients with advanced NSCLC. Nineteen individuals were enrolled and stratified into R and NR groups based on their baseline characteristics. A significant difference in BMI was observed between the two groups (Table 1), which indicated that BMI can affect prognosis after ICI treatment. BMI may influence the effectiveness of ICI therapy in patients with advanced NSCLC, and patients with a high BMI may experience longer PFS and OS than those with a low BMI^37,38^. Consequently, patients with advanced NSCLC and a low BMI may have a poorer prognosis when undergoing ICI therapy.

Initially, our study revealed that the patients in the R group exhibited elevated a-diversity in their fecal microbiome, as determined by both the Shannon index and Faith’s PD (Table 2). In particular, individuals who responded positively to ICI treatment possess a diverse gut microbiota, which is consistent with our findings^39^.

The analysis of the intestinal microbiota via NGS allowed to stratify patients according to R and NR groups and revealed that the abundance of *Bifidobacteriaceae* was significantly higher in the R group. In other words, the results suggest that ICI treatment may be more effective in patients with more indigenous bifidobacteria (Table 3). Previous studies have suggested that *Bifidobacterium* is more abundant in ICI treatment responders than in non-responders^14,40^. This implies that *Bifidobacterium* plays a crucial role in enhancing the antitumor efficacy of ICIs. There was no significant difference in PD-L1-positive rate/ PD-L1-expression rate between the high and low groups of a-diversities and relative abundance of Bifidobacteriaceae (Supplementary Table S3). Thus, the possibility of a relationship between the intestinal microbiota and PD-L1 expression was ruled out.

In addition to the NGS results, the results of the gut microbiota analysis by RT-qPCR for the R and NR groups showed that the abundance of *L. brevis* was significantly higher in the R group (Table 4). Univariate analysis demonstrated that a high abundance of *Bifidobacteriaceae* and/or the presence of *L. brevis* in the gut microbiota significantly reduced the risk of death during ICI therapy. Log-rank analyses of PFS and OS suggested that the presence of *Bifidobacteriaceae* and/or *L. brevis* in the gut microbiota before administration of ICI may contribute to prolonged OS (Fig. 1). *Bifidobacterium* improves the effectiveness of anti-PD-L1 antibodies^15,40^, leading to prolonged OS in patients with high *Bifidobacteriaceae* abundance. In fact, the relative abundance of *Bifidobacteriaceae* in patients in the “Yes” group, who received antibiotics prior to treatment, was significantly lower than that in patients in the “No” group (Supplementary Table S5). The effect of antibiotic dysbiosis was also observed in terms of *L. brevis* counts (Supplementary Table S6). Notably, the risk of death was significantly higher in the “Yes” group than in the “No” group (Table 5), and fewer antibiotic users were in the R group than in the NR group (Table 1).

In the analysis of the relationship between clinical and microenvironmental parameters of the gut and prognosis, the risk of death was significantly increased in the antibiotic “Yes” group. However, the risk of disease progression was not significant (Table 5). Since PFS is the duration until NSCLC progression, and OS is the duration until death caused by not only NSCLC but also all other reasons, they do not always fluctuate in the same manner. In the case of antibiotics especially, the risk was significantly increased in the antibiotic “Yes” group, because patients’ condition was bad and they might also have other disease, NSCLC progression itself might not be the cause of death.

Although antibiotics are essential for the treatment of tract infections and pneumonia, their use before starting ICI treatment likely disrupts the intestinal environment, affects the abundance of *Bifidobacteriaceae*, and reduces the efficacy of ICI treatments. Consistent with our results, Derosa et al. reported that antibiotic use reduced the therapeutic efficacy of ICIs^41^. The antibiotic usage prior to at least 6 months before the sample collection would be more appropriate to document. However, it was thought to be difficult to collect a sufficient number of patients who did not use antibiotics during the 6 months before sample collection in the NCSCL. Although the study did not evaluate changes in the gut microbiota due to antibiotic use, it suggested that the recovery and alleviation of gut microbiota dysbiosis, particularly the abundance of *Bifidobacteriaceae*, is important.

This study demonstrated that a high occupancy rate of bifidobacteria may be advantageous in ICI treatment. Nonetheless, a review of prior reports has indicated that bifidobacteria may exert a negative impact on ICI treatment^17,18^. We achieved high accuracy in detecting bifidobacteria using 16S rRNA V1-2 sequencing; however, as noted in the limitations, we did not investigate the underlying mechanism. Short-chain fatty acids, the primary metabolites of bifidobacteria, have been reported to exert diverse physiological effects ranging from the intestinal tract to the entire body; however, their contribution to the therapeutic effects of ICI remains unknown^42,43^. Although we did not examine bifidobacteria metabolites in this study, the discrepancy in occupancy rate comparisons may be elucidated by examining the mutual interactions with other coexisting microorganisms through an analysis of the functional diversity of the intestinal microbiota, including the effects of metabolites^18^.

In the present study, we identified *L. brevis* as a novel bacterium that may enhance the efficacy of ICIs. Patients who tested positive for *L. brevis* exhibited significantly longer PFS and OS than those who tested negative for *L. brevis* (Fig. 1b). Previous studies have reported anti-inflammatory^44,45^ and immunostimulatory^46^ effects of *L. brevis*; however, this study is the first to establish a connection between *L. brevis* and the efficacy of ICI treatment. The number of *L. brevis* in this species is typically low in the gut microbiota, with a low detection rate^47–49^. However, in this study, the detection rate for *L. brevis* was high (6/17 patients). We did not investigate the history of food consumption of the participants; therefore, we cannot explain the high detection rate of *L. brevis*, especially in the R group (6/10 patients). Notably, Osaka, where this study was conducted, is situated near Kyoto, where *L. brevis*-rich pickles are traditionally consumed. Therefore, further investigation in a larger cohort is necessary to clarify the relationship between *L. brevis* and ICI treatment, considering the regional food culture.

Our study has certain limitations in terms of scope. Therefore, a larger sample size is necessary for a more comprehensive examination. Thus, additional large-scale studies are required to corroborate the role of *Bifidobacteriaceae* and *L. brevis* in the context of ICI therapy. We have no information that makes it possible to speculate on the mechanism of the role of the microbiota in the efficacy of ICI.

In conclusion, the key findings of this observational study revolved around the significance of the gut microbiome in patients with advanced NSCLC receiving ICI therapy. Specifically, the diversity and abundance of *Bifidobacteriaceae* in the fecal microbiomes of responders were higher than those of non-responders. Furthermore, our study highlights the potential adverse effects of antibiotics and the positive influence of *L. brevis* on therapy. Based on our findings, it is crucial to develop an effective strategy for maintaining a healthy gut microenvironment, particularly before and during ICI treatment, even when antibiotics are used. For instance, supplementation with *Bifidobacterium* and *L. brevis* prior to antibiotic treatment may mitigate the decline in indigenous *Bifidobacterium* and *L. brevis* populations and may enhance the effects of ICI treatment. It is essential to validate the efficacy of *Bifidobacterium* and *L. brevis* in large-scale studies, including controlled clinical trials.

## Supplementary Information

Supplementary Information.

## Data Availability

The fecal 16S rRNA gene amplicon sequencing datasets are available in the Sequence Read Archive of the National Center for Biotechnology Information under accession number PRJNA1203384, https://www.ncbi.nlm.nih.gov/bioproject/PRJNA1203384. The author declares that all other datasets generated during and/or analyzed during the current study are available in this article and its Supplementary file, or from the corresponding author on reasonable request.

## References

[1] Thai, A. A., Solomon, B. J., Sequist, L. V., Gainor, J. F. & Heist, R. S. Lung cancer. *Lancet* **398**, 535–554 (2021). 34273294 10.1016/S0140-6736(21)00312-3

[2] Svoboda, E. Artificial intelligence is improving the detection of lung cancer. *Nature* **587**, S20–S22 (2020). 33208974 10.1038/d41586-020-03157-9

[3] Cancer statistics in Japan 2023. *Statistics*, pdf. https://ganjoho.jp/public/qa_links/report/statistics/pdf/Cancer. (2023).

[4] Mazieres, J. et al. Immune checkpoint inhibitors for patients with advanced lung cancer and oncogenic driver alterations: results from the immunotarget registry. *Ann. Oncol.* **30**, 1321–1328 (2019). 31125062 10.1093/annonc/mdz167 PMC7389252

[5] Vokes, E. E. et al. Nivolumab versus docetaxel in previously treated advanced non-small-cell lung cancer (CheckMate 017 and CheckMate 057): 3-year update and outcomes in patients with liver metastases. *Ann. Oncol.* **29**, 959–965 (2018). 29408986 10.1093/annonc/mdy041

[6] Paz-Ares, L. et al. Pembrolizumab plus chemotherapy for squamous non-small-cell lung cancer. *N. Engl. J. Med.* **379**, 2040–2051 (2018). 30280635 10.1056/NEJMoa1810865

[7] Barnet, M. B., Cooper, W. A., Boyer, M. J. & Kao, S. Immunotherapy in non-small cell lung cancer: shifting prognostic paradigms. *J. Clin. Med.* **14**, 151 (2018). 10.3390/jcm7060151 PMC6024943 29904031

[8] Huang, Q. et al. Non-small cell lung cancer in the era of immunotherapy. *Semin. Oncol.* **49**, 337–343 (2022). 35879123 10.1053/j.seminoncol.2022.06.009

[9] Vétizou, M. et al. Anticancer immunotherapy by CTLA-4 blockade relies on the gut microbiota. *Science* **350**, 1079–1084 (2015). 26541610 10.1126/science.aad1329 PMC4721659

[10] Iida, N. et al. Commensal bacteria control cancer response to therapy by modulating the tumor microenvironment. *Science* **342**, 967–970 (2013). 24264989 10.1126/science.1240527 PMC6709532

[11] Sivan, A. et al. Commensal Bifidobacterium promotes antitumor immunity and facilitates anti-PD-L1 efficacy. *Science* **350**, 1084–1089 (2015). 26541606 10.1126/science.aac4255 PMC4873287

[12] Pierrard, J. & Seront, E. Impact of the gut microbiome on immune checkpoint inhibitor efficacy-a systematic review. *Curr. Oncol.* **26**, 395–403 (2019). 31896938 10.3747/co.26.5177 PMC6927774

[13] Chaput, N. et al. Baseline gut microbiota predicts clinical response and colitis in metastatic melanoma patients treated with ipilimumab. *Ann. Oncol.* **28**, 1368–1379 (2017). 28368458 10.1093/annonc/mdx108

[14] Routy, B. et al. Gut microbiome influences efficacy of PD-1-based immunotherapy against epithelial tumors. *Science* **359**, 91–97 (2018). 29097494 10.1126/science.aan3706

[15] Jin, Y. et al. The diversity of gut microbiome is associated with favorable responses to anti-programmed death 1 immunotherapy in Chinese patients with NSCLC. *J. Thorac. Oncol.* **14**, 1378–1389 (2019). 31026576 10.1016/j.jtho.2019.04.007

[16] Hakozaki, T. et al. The gut microbiome associates with immune checkpoint inhibition outcomes in patients with advanced non-small cell lung cancer. *Cancer Immunol. Res.* **8**, 1243–1250 (2020). 32847937 10.1158/2326-6066.CIR-20-0196

[17] Dora, D. et al. Non-small cell lung cancer patients treated with anti-PD1 immunotherapy show distinct microbial signatures and metabolic pathways according to progression-free survival and PD-L1 status. *Oncoimmunology* **12**, 2204746 (2023). 37197440 10.1080/2162402X.2023.2204746 PMC10184596

[18] Dora, D. et al. From bench to bedside: an interdisciplinary journey through the gut-lung axis with insights into lung cancer and immunotherapy. *Front. Immunol.* **15**, 1434804 (2024). 39301033 10.3389/fimmu.2024.1434804 PMC11410641

[19] Syromyatnikov, M. et al. Characteristics of the gut bacterial composition in people of different nationalities and religions. *Microorganisms* **10**, 1866 (2022). 36144468 10.3390/microorganisms10091866 PMC9501501

[20] Kato, K. et al. Association between functional lactase variants and a high abundance of Bifidobacterium in the gut of healthy Japanese people. *PLoS ONE* **13**, e0206189 (2018). 30339693 10.1371/journal.pone.0206189 PMC6195297

[21] Takagi, T. et al. Typing of the gut microbiota community in Japanese subjects. *Microorganisms* **10**, 664 (2022). 35336239 10.3390/microorganisms10030664 PMC8954045

[22] Kubota, H. et al. Detection of human intestinal catalase-negative, gram-positive cocci by rRNA-targeted reverse transcription-PCR. *Appl. Environ. Microbiol.* **76**, 5440–5451 (2010). 20581195 10.1128/AEM.03132-09 PMC2918946

[23] Matsuki, T., Watanabe, K., Fujimoto, J., Takada, T. & Tanaka, R. Use of 16S rRNA gene-targeted group-specific primers for real-time PCR analysis of predominant bacteria in human feces. *Appl. Environ. Microbiol.* **70**, 7220–7228 (2004). 15574920 10.1128/AEM.70.12.7220-7228.2004 PMC535136

[24] Gonai, M. et al. Galacto-oligosaccharides ameliorate dysbiotic Bifidobacteriaceae decline in Japanese patients with type 2 diabetes. *Benef. Microbes* **8**, 705–716 (2017). 28884590 10.3920/BM2016.0230

[25] Ikeda, T. et al. Alteration in faecal bile acids, gut microbial composition and diversity after laparoscopic sleeve gastrectomy. *Br. J. Surg.* **107**, 1673–1685 (2020). 32432347 10.1002/bjs.11654

[26] Bolyen, E. et al. Reproducible, interactive, scalable and extensible microbiome data science using QIIME 2. *Nat. Biotechnol.* **37**, 852–857 (2019). 31341288 10.1038/s41587-019-0209-9 PMC7015180

[27] Matsuda, K., Tsuji, H., Asahara, T., Kado, Y. & Nomoto, K. Sensitive quantitative detection of commensal bacteria by rRNA-targeted reverse transcription-PCR. *Appl. Environ. Microbiol.* **73**, 32–39 (2007). 17071791 10.1128/AEM.01224-06 PMC1797142

[28] Matsuda, K. et al. Establishment of an analytical system for the human fecal microbiota, based on reverse transcription-quantitative PCR targeting of multicopy rRNA molecules. *Appl. Environ. Microbiol.* **75**, 1961–1969 (2009). 19201979 10.1128/AEM.01843-08 PMC2663197

[29] Sakaguchi, S. et al. Bacterial rRNA-targeted reverse transcription-PCR used to identify pathogens responsible for fever with neutropenia. *J. Clin. Microbiol.* **48**, 1624–1628 (2010). 20351213 10.1128/JCM.01724-09 PMC2863901

[30] Ohigashi, S. et al. Changes of the intestinal microbiota, short chain fatty acids, and fecal pH in patients with colorectal cancer. *Dig. Dis. Sci.* **58**, 1717–1726 (2013). 23306850 10.1007/s10620-012-2526-4

[31] Matsuki, T. et al. Development of 16S rRNA-gene-targeted group-specific primers for the detection and identification of predominant bacteria in human feces. *Appl. Environ. Microbiol.* **68**, 5445–5451 (2002). 12406736 10.1128/AEM.68.11.5445-5451.2002 PMC129894

[32] Matsuki, T. Development of quantitative PCR detection method with 16S rRNA gene-targeted genus- and species-specific primers for the analysis of human intestinal microflora and its application. *Nihon Saikingaku Zasshi* **62**, 255–261 (2007). 17575792 10.3412/jsb.62.255

[33] Kurakawa, T. et al. Establishment of a sensitive system for analysis of human vaginal microbiota on the basis of rRNA-targeted reverse transcription-quantitative PCR. *J. Microbiol. Methods* **111**, 93–104 (2015). 25661498 10.1016/j.mimet.2015.01.021

[34] Matsuki, T., Watanabe, K., Tanaka, R., Fukuda, M. & Oyaizu, H. Distribution of bifidobacterial species in human intestinal microflora examined with 16S rRNA-gene-targeted species-specific primers. *Appl. Environ. Microbiol.* **65**, 4506–4512 (1999). 10508082 10.1128/aem.65.10.4506-4512.1999 PMC91600

[35] Matsuda, K. et al. Sensitive quantification of clostridium difficile cells by reverse transcription-quantitative PCR targeting rRNA molecules. *Appl. Environ. Microbiol.* **78**, 5111–5118 (2012). 22582062 10.1128/AEM.07990-11 PMC3416433

[36] Kikuchi, E., Miyamoto, Y., Narushima, S. & Itoh, K. Design of species-specific primers to identify 13 species of Clostridium harbored in human intestinal tracts. *Microbiol. Immunol.* **46**, 353–358 (2002). 12139395 10.1111/j.1348-0421.2002.tb02706.x

[37] Ichihara, E. et al. The impact of body mass index on the efficacy of anti-PD-1/PD-L1 antibodies in patients with non-small cell lung cancer. *Lung Cancer* **139**, 140–145 (2020). 31786476 10.1016/j.lungcan.2019.11.011

[38] Gelibter, A. et al. Status of correlation between BMI and response to immunocheck-point inhibitor in advanced non-small-cell lung cancer. *Lung Cancer Manag.* **9**, LMT26 (2020). 32346402 10.2217/lmt-2019-0016 PMC7186849

[39] Gopalakrishnan, V. et al. Gut microbiome modulates response to anti-PD-1 immunotherapy in melanoma patients. *Science* **359**, 97–103 (2018). 29097493 10.1126/science.aan4236 PMC5827966

[40] Matson, V. et al. The commensal microbiome is associated with anti-PD-1 efficacy in metastatic melanoma patients. *Science* **359**, 104–108 (2018). 29302014 10.1126/science.aao3290 PMC6707353

[41] Derosa, L. et al. Negative association of antibiotics on clinical activity of immune checkpoint inhibitors in patients with advanced renal cell and non-small-cell lung cancer. *Ann. Oncol.* **29**, 1437–1444 (2018). 29617710 10.1093/annonc/mdy103 PMC6354674

[42] Fusco, W. et al. Short-chain fatty-acid-producing bacteria: key components of the human gut microbiota. *Nutrients* **15**, 2211 (2023). 37432351 10.3390/nu15092211 PMC10180739

[43] Markowiak-Kopeć, P. & Śliżewska, K. The effect of probiotics on the production of short-chain fatty acids by human intestinal microbiome. *Nutrients* **12**, 1107 (2020). 32316181 10.3390/nu12041107 PMC7230973

[44] Ding, S. et al. Lactobacillus brevis alleviates DSS-induced colitis by reprograming intestinal microbiota and influencing serum metabolome in murine model. *Front. Physiol.* **10**, 1152 (2019). 31620010 10.3389/fphys.2019.01152 PMC6759783

[45] Sharma, A. et al. Lactobacillus brevis CD2 lozenges reduce radiation- and chemotherapy-induced mucositis in patients with head and neck cancer: a randomized double-blind placebo-controlled study. *Eur. J. Cancer* **48**, 875–881 (2012). 21741230 10.1016/j.ejca.2011.06.010

[46] Jeong, M. et al. Heat-killed Lactobacillus brevis enhances phagocytic activity and generates immune-stimulatory effects through activating the TAK1 pathway. *J. Microbiol. Biotechnol.* **30**, 1395–1403 (2020). 32627755 10.4014/jmb.2002.02004 PMC9728231

[47] Suzuki, Y. et al. Association between yogurt consumption and intestinal microbiota in healthy young adults differs by host gender. *Front. Microbiol.* **8**, 847 (2017). 28553274 10.3389/fmicb.2017.00847 PMC5425481

[48] Shimizu, K. et al. Synbiotics modulate gut microbiota and reduce enteritis and ventilator-associated pneumonia in patients with sepsis: a randomized controlled trial. *Crit. Care* **22**, 239 (2018). 30261905 10.1186/s13054-018-2167-x PMC6161427

[49] Kanazawa, A. et al. Effects of synbiotic supplementation on chronic inflammation and the gut microbiota in obese patients with type 2 diabetes mellitus: a randomized controlled study. *Nutrients* **13**, 558 (2021). 33567701 10.3390/nu13020558 PMC7914668

