# Supplementary material for: Characteristics of the gut microbiota in patients with advanced non-small cell lung cancer who responded to immune checkpoint inhibitors
Source: Sci Rep. 2025 Jul 2;15:23398. doi: 10.1038/s41598-025-08049-4 (PMC12222521; doi:10.1038/s41598-025-08049-4)
Supplement: Supplementary file 1 — Supplementary Information. [file 41598_2025_8049_MOESM1_ESM.pdf]

Characteristics of the gut microbiota in patients with advanced non-small cell lung cancer who responded to immune checkpoint inhibitors

Hideyuki Komatsu[1], Takuya Sugimoto[1], Yuzuru Ogata[1], Takahito Miura[1], Masanori Aida[1], Hiroyuki Nishiyama[1], Mitsuhisa Kawai[1], Yukihiro Yano[2], Masahide Mori[2], and Yoshiyuki Shishido[1*]

[1]Yakult Central Institute, Yakult Honsha Co., Ltd., Tokyo 186-8650, Japan

[2]Department of Thoracic Oncology, National Hospital Organization, NHO Osaka Toneyama Medical Center, 5-1-1 Toneyama, Toyonaka-shi, Osaka 560-8552, Japan

***Corresponding author**

Yoshiyuki Shishido

Yakult Central Institute, 5-11 Izumi, Kunitachi-shi, Tokyo 186-8650, Japan

Tel.: +81-042-577-8960; Fax: +81-042-577-3020; E-mail: yoshiyuki-shishido@yakult.co.jp

Table S1. Sequences of the primers used in determination of the bacterial count by rRNA-targeted reverse transcription-quantitative PCR

Target bacteria	Primer	Sequence (5′ - 3′)	Reference
Clostridium coccoides group	g-Ccoc-F	AAATGACGGTACCTGACTAA	28
	g-Ccoc-R	CTTTGAGTTTCATTCTTGCGAA	28
Clostridium leptum subgroup	sg-Clept-F	GCACAAGCAGTGGAGT	20
	sg-Clept-R3	CTTCCTCCGTTTTGTCAA	20
Bacteroides fragilis group	g-Bfra-F2	AYAGCCTTTCGAAAGRAAGAT	29
	g-Bfra-R	CCAGTATCAACTGCAATTTTA	28
Bifidobacterium	g-Bifid-F	CTCCTGGAAACGGGTGG	28
	g-Bifid-R	GGTGTTCTTCCCGATATCTACA	28
Bifidobacterium longum subsp. longum	BLON-23S-F	TGGGACAGGCAAATCCGTC	30
	BLON-23S-R	TTTGAGCCTGACGCCGAAGC	30
Bifidobacterium breve	BiBRE-1	CCGGATGCTCCATCACAC	31
	BiBRE-2	ACAAAGTGCCTTGCTCCCT	31
Bifidobacterium bifidum	BiBIF-1	CCACATGATCGCATGTGATTG	31
	BiBIF-2	CCGAAGGCTTGCTCCCAAA	31
Bifidobacterium catenulatum group	BiCATg-1	CGGATGCTCCGACTCCT	31
	BiCATg-2	CGAAGGCTTGCTCCCGAT	31
Bifidobacterium adolescentis	BADO-23S-F	AGCAATCTTCATGGTTGC	30
	BADO-23S-R	ACCGTCTCGGTTTTGCCGGTCCATG	30
Atopobium cluster	g-Atopo-F	GGGTTGAGAGACCGACC	20
	g-Atopo-R	CGGRGCTTCTTCTGCAGG	20
Prevotella	g-Prevo-F	CACRGTAAACGATGGATGCC	28
	g-Prevo-R	GGTCGGGTTGCAGACC	28
Clostridioides difficile	Cd-lsu-F	GGGAGCTTCCCATACGGGTTG	32
(formerly *Clostridium difficile*)	Cd-lsu-R	TTGACTGCCTCAATGCTTGGGC	32
Clostridium perfringens	s-Clper-F	GGGGGTTTCAACACCTCC	33
	ClPER-R	GCAAGGGATGTCAAGTGT	20
Lactobacillus	sg-Lgas-F	GATGCATAGCCGAGTTGAGAGACTGAT	25
(formerly *Lactobacillus gasseri* subgroup)	sg-Lgas-R	TAAAGGCCAGTTACTACCTCTATCC	25
Levilactobacillus brevis	s-Lbre-F	ATTTTGTTTGAAAGGTGGCTTCGG	25
(formerly *Lactobacillus brevis*)	s-Lbre-R	ACCCTTGAACAGTTACTCTCAAAGG	25
Lacticaseibacillus	sg-Lcas-F	ACCGCATGGTTCTTGGC	25
(formerly *Lactobacillus casei* subgroup)	sg-Lcas-R	CCGACAACAGTTACTCTGCC	25
Limosilactobacillus fermentum	LFer-1	CCTGATTGATTTTGGTCGCCAAC	25
(formerly *Lactobacillus fermentum*)	LFer-2	ACGTATGAACAGTTACTCTCATACGT	25
Fructilactobacillus fructivorans	s-Lfru-F	TGCGCCTAATGATAGTTGA	25
(formerly *Lactobacillus fructivorans*)	s-Lfru-R	GATACCGTCGCGACGTGAG	25
Lactiplantibacillus	sg-Lpla-F	CTCTGGTATTGATTGGTGCTTGCAT	25
(formerly *Lactobacillus plantarum* subgroup)	sg-Lpla-R	GTTCGCCACTCACTCAAATGTAAA	25
Limosilactobacillus (except *L. fermentum*)	sg-Lreu-F	GAACGCAYTGGCCCAA	25
(formerly *Lactobacillus reuteri* subgroup)	sg-Lreu-R	TCCATTGTGGCCGATCAGT	25
Liquorilactobacillus and *Ligilactobacillus*	sg-Lrum-F	CACCGAATGCTTGCAYTCACC	25
(formerly *Lactobacillus ruminis* subgroup)	sg-Lrum-R	GCCGCGGGTCCATCCAAAA	25
Latilactobacillus	sg-Lsak-F	CATAAAACCTAMCACCGCATGG	25
(formerly *Lactobacillus sakei* subgroup)	sg-Lsak-R	TCAGTTACTATCAGATACRTTCTTCTC	25

Table S1. *Cont.*

Target bacteria	Primer	Sequence (5′- 3′)	Reference
Enterobacteriaceae	En-lsu-3F	TGCCGTAACTTCGGGAGAAGGCA	24
	En-lsu-3'R	TCAAGGACCAGTGTTCAGTGTC	24
Enterococcus	g-Encoc-F	ATCAGAGGGGGATAACACTT	25
	g-Encoc-R	ACTCTCATCCTTGTTCTTCTC	25
Streptococcus	g-Str-F	AGCTTAGAAGCAGCTATTCATTC	26
	g-Str-R	GGATACACCTTTCGGTCTCTC	26
Staphylococcus	g-Staph-F	TTTGGGCTACACACGTGCTACAATGGACAA	25
	g-Staph-R	AACAACTTTATGGGATTTGCWTGA	25
Pseudomonas	PSD7F	CAAAACTACTGAGCTAGAGTACG	24
	PSD7R	TAAGATCTCAAGGATCCCAACGGCT	24

Group-, genus- or species-specific primer sets were developed by using 16S rDNA sequences, except for Cd-lsu-F/R, En-lsu-3F/3 ′R, and g-Str-F/R, which targeted 23S rDNA.

Table S2. Differences in relative abundances of the R and NR groups by 16S rRNA sequencing.

A. At the phylum level

Phylum	Relative abundance (%)				*p*-value
	R		NR		
Actinobacteriota	5.32	[1.73, 11.84]	1.46	[1.11, 5.18]	0.196
Bacteroidota	32.90	[23.83, 52.00]	48.61	[40.13, 51.15]	0.432
Desulfobacterota	0.11	[0.03, 0.20]	0.11	[0.04, 0.14]	0.553
Firmicutes	49.72	[39.94, 64.19]	47.82	[41.81, 56.58]	0.902
Proteobacteria	1.71	[0.28, 2.07]	1.27	[0.73, 3.11]	0.773

R, responder; NR, non-responder. Data are displayed as median [interquartile range]. *p*-values were estimated using the Mann–Whitney *U* test.

B. At the genus level

Genus	Relative abundance (%)				*p*-value
	R		NR		
Bifidobacterium	2.94	[0.77, 9.83]	0.18	[0.03, 1.22]	**0.045**
Faecalibacterium	1.91	[0.36, 3.76]	1.32	[0.01, 2.81]	0.583
Incertae Sedis	0.23	[0.12, 0.45]	0.07	[0.03, 0.38]	0.167
Ruminococcus	1.98	[1.17, 4.00]	0.00	[0.00, 1.08]	**0.035**
Subdoligranulum	0.56	[0.02, 2.54]	0.00	[0.00, 0.36]	0.100
uncultured	0.24	[0.08, 0.36]	0.08	[0.06, 0.09]	0.167

R, responder; NR, non-responder. Data are displayed as median [interquartile range]. *p*-values were estimated using the Mann–Whitney *U* test. *p*-value entries in bold indicate $p < 0.05$.

Table S3. Differences in the PD-L1 expression of high and low groups of α-diversities and relative abundance of *Bifidobacteriaceae*

Variables		Shannon Index		*p*-value	Faith's PD		*p*-value	*Bifidobacteriaceae*		*p*-value
		High (10)	Low (9)		High (9)	Low (10)		High (9)	Low (10)	
PD-L1 expression,	<50%/≥50%	2 / 8	5 / 4	0.170	4 / 5	3 / 7	0.650	2 / 7	5 / 5	0.350
	0%/≥1%	1 / 9	2 / 7	0.582	1 / 8	2 / 8	1.000	1 / 8	2 / 8	1.000

p-values were estimated using Fisher's exact test. The post-hoc statistical power at a significance level of $p < 0.05$ was calculated as follows: for PD-L1 expression <50%/≥50%, the Shannon Index, Faith's PD, and *Bifidobacteriaceae* were 15.2%, not a number (NaN), and 7.8%, respectively. For PD-L1 expression 0%/≥1%, the Shannon Index, Faith's PD, and *Bifidobacteriaceae* were all NaN.

Table S4. Detection frequency of *L. brevis* between the R and NR groups.

Group	*L. brevis*-positive	*L. brevis*-negative		*p*-value
R	6	4	10	**0.035**
NR	0	7	7	
	6	11	17	

R, responder; NR, non-responder. *p*-value was estimated using Fisher's exact test. *p*-value entries in bold indicate $p < 0.05$. The post-hoc statistical power at a significance level of $p < 0.05$ was calculated as 59.9%.

Table S5. Relative abundances of gut microbiota between antibiotics "No" group and antibiotics "Yes" group at the family level determined by 16S rRNA sequencing.

Family	Antibiotics		*p*-value
	No (n = 11)	Yes (n =8)	
Bifidobacteriaceae	4.11 [1.73, 10.85]	0.17 [0.02, 0.75]	**0.005**
Christensenellaceae	0.09 [0.03, 1.07]	0.05 [0.03, 0.10]	0.281
Ruminococcaceae	7.00 [3.43, 11.13]	3.76 [1.48, 8.15]	0.238

The data indicate in family with median abundance rate of over 0.1%. Data are expressed as median [interquartile range]. *p*-values were estimated using the Mann–Whitney *U* test. *p*-value entries in bold indicate $p < 0.05$.

Table S6. Effect of antibiotics on the count of *L. brevis* as measured by RT-qPCR.

Genus	Antibiotics		*p*-value
	No (n = 9)	Yes (n =8)	
L. brevis	3.04 [1.15, 3.93]	1.15 [1.15, 1.15]	**0.043**

Data are expressed as median [interquartile range]. *p*-value was estimated using the Mann–Whitney *U* test. *p*-value entries in bold indicate $p < 0.05$.
